# Improving our understanding of the environmental persistence of chemicals

**DOI:** 10.1002/ieam.4438

**Published:** 2021-05-25

**Authors:** Graham Whale, John Parsons, Kees van Ginkel, Russell Davenport, Eleni Vaiopoulou, Kathrin Fenner, Andreas Schaeffer

**Affiliations:** ^1^ Whale Environmental Consultancy Ltd. Chester UK; ^2^ Institute for Biodiversity and Ecosystem Dynamics University of Amsterdam Amsterdam The Netherlands; ^3^ GinkelBiodeg Wageningen The Netherlands; ^4^ School of Engineering Newcastle University Newcastle upon Tyne UK; ^5^ Concawe Brussels Belgium; ^6^ Chemistry Department University of Zürich Zürich Switzerland; ^7^ Institute for Environmental Research Aachen University Aachen Germany

**Keywords:** Biodegradation, Environmental risk assessment, Persistence, REACH, Screening

## Abstract

Significant progress has been made in the scientific understanding of factors that influence the outcome of biodegradation tests used to assess the persistence (P) of chemicals. This needs to be evaluated to assess whether recently acquired knowledge could enhance existing regulations and environmental risk assessments. Biodegradation tests have limitations, which are accentuated for “difficult‐to‐test” substances, and failure to recognize these can potentially lead to inappropriate conclusions regarding a chemical's environmental persistence. Many of these limitations have been previously recognized and discussed in a series of ECETOC reports and workshops. These were subsequently used to develop a series of research projects designed to address key issues and, where possible, propose methods to mitigate the limitations of current assessments. Here, we report on the output of a Cefic LRI–Concawe Workshop held in Helsinki on September 27, 2018. The objectives of this workshop were to disseminate key findings from recent projects and assess how new scientific knowledge can potentially support and improve assessments under existing regulatory frameworks. The workshop provided a unique opportunity to initiate a process to reexamine the fundamentals of degradation and what current assessment methods can achieve by (1) providing an overview of the key elements and messages coming from recent research initiatives and (2) stimulating discussion regarding how these interrelate and how new findings can be developed to improve persistence assessments. Opportunities to try and improve understanding of factors affecting biodegradation assessments and better understanding of the persistence of chemicals (particularly UVCBs [substances of unknown or variable composition, complex reaction products, or biological materials]) were identified, and the workshop acted as a catalyst for further multistakeholder activities and engagements to take the persistence assessment of chemicals into the 21st century. *Integr Environ Assess Manag* 2021;17:1123–1135. © 2021 European Petroleum Refiners Association – Concawe Division. *Integrated Environmental Assessment and Management* published by Wiley Periodicals LLC on behalf of Society of Environmental Toxicology & Chemistry (SETAC).

## KEY POINTS

- A multistakeholder workshop discussed implications of recent developments in biodegradation tests used to assess persistence of chemicals.
- Recognition that current tests and approaches to assess chemical persistence have limitations, especially for difficult‐to‐test substances.
- Opportunities to try and improve understanding of factors affecting biodegradation assessments and better understanding of the persistence of chemicals (particularly UVCBs) were identified.
- The workshop acted as a catalyst for further multistakeholder activities and engagements to take the persistence assessment of chemicals into the 21st century.

## INTRODUCTION

The environmental hazard assessment of chemicals is based primarily on their persistence (P), bioaccumulation (B), and toxicity (T), the so‐called PBT properties. International chemical regulations incorporate guidelines and methods to assess these properties. For example, under the European Union Registration, Evaluation, Authorisation and Restriction of Chemicals (EU REACH) regulations, the European Chemicals Agency (ECHA) provides guidance (ECHA, 2017), which is periodically reviewed and revised. The ECHA requires a PBT/vPvB (very Persistent and very Bioaccumulative) assessment to be performed for all substances for which a chemical safety assessment must be conducted. In effect, these data are required for all substances manufactured or imported in the European Union in amounts above 10 tonnes/year that are not specifically exempted (e.g., naturally occurring substances) from registration. The ECHA PBT/vPvB assessment has been developed to determine in a stepwise manner if a substance fulfills the criteria specified in Annex XIII of the regulation.

Persistence of chemicals in the environment is regarded as a cornerstone of chemical assessment, because this influences potential for exposure and has historical significance as a key parameter for estimating the risk of long‐term adverse effects on biota (Matthies et al., 2016). This field of science and accompanying regulations were developed due to growing concerns regarding events, such as foaming in rivers in the 1950s (due to nonreadily biodegradable surfactants such as alkyl benzene sulfonates (Sallee et al., 1956) and advances in chemical analysis in the 1960s, which led to the detection of persistent and bioaccumulative chemicals in Arctic and Antarctic mammals. These concerns led to the development of the United Nations Environment Programme (UNEP) Stockholm Convention on persistent organic pollutants (POPs), which was adopted in 2001, entered into force in 2004, and last revised in 2017 (UNEP, 2018). In addition, there are growing concerns regarding persistent mobile organic compounds (PMT concept), which may enter drinking water (Reemtsma et al., 2016). The claim has been made that high persistence alone should be established as a sufficient basis for regulation of a chemical, the so‐called “P‐sufficient approach” (Cousins et al., 2019). If such approaches are to be adopted, they will need to be underpinned by weight of evidence assessments that clearly identify environmental persistence, alleviating concerns that these are simply artifacts of the current testing strategy. This is an important consideration because concerns have been raised that problems could arise if existing tests, regulations, and guidance for persistence assessment become outdated (Whale et al., 2018). It is recognized that there is a recurring need to update the existing test guidelines, revisit regulatory approaches, and update related guidance documents. As such, the workshop formed part of a strategy to help ensure that scientific advances in persistence assessment are evaluated and, where appropriate, ultimately lead to relevant updates.

Due to the criticality of persistence assessments, the European Chemical Industry Council (Cefic) has actively funded research on this issue since 1999 as one of the first of its Long‐Range Research Initiative (LRI) programs (Cefic, 1999). Along with this research initiative, the European Centre for Ecotoxicology and Toxicology of Chemicals (ECETOC) has also hosted a number of stakeholder workshops on biodegradation and persistence assessment (ECETOC, 2003, 2007, 2012). The emphasis of these workshops is to disseminate new research findings as well as identify further research and concepts to better understand the key limitations of existing tests and approaches with a view to propose new approaches to mitigate these limitations.

In September 2018, a Cefic LRI–Concawe Workshop entitled “Initiative towards improving our understanding of persistence in the 21st century” was held in Helsinki. Concawe (Conservation of Clean Air and Water in Europe) supported this initiative due to its interest and recent sponsored research into methods to assess the persistence of constituents of petroleum substances. Petroleum substances are composed of a vast number (many thousands) of unique hydrocarbons that each exhibit different properties relevant to environmental assessment, and as such are so‐called UVCBs (substances of unknown or variable composition, complex reaction products, or biological materials). Due to their complex composition, the assessment of UVCBs presents significant challenges when determining biodegradation potential and environmental persistence for regulatory purposes (Brown et al., 2020). Consequently, standard assessment methods may not be applicable for many petroleum and difficult‐to‐test substances and new scientific methods and risk assessment approaches are required. This is because, historically, product risk assessment and even test methods have predominantly been developed for substances with unique properties (e.g., crop protection products, surfactants, biocides) and as such may not be directly applicable to all chemical substances. This is recognized in the Organisation for Economic Co‐operation and Development (OECD) guidelines for the testing of degradation of organic chemicals (OECD, 2006), which state, for example, that ready biodegradability tests are intended for pure substances and are generally not applicable for complex compositions containing different types of constituents, like UVCBs.

The workshop was organized to provide an opportunity to disseminate recent research, assess how communication and awareness of recent developments could be improved, and initiate discussions on elements that could potentially be used to enhance regulatory persistence assessment. It also provided an opportunity to understand some of the concerns and challenges facing the different academic, regulatory, and industrial communities working in this field. This was seen as an important step to develop some core ideas for how to translate key knowledge from past and ongoing research into recommendations to be considered in any updates in the regulatory assessment of persistence.

Presentations at the workshop were predominantly based on available recent Cefic and Concawe sponsored work, and the workshop was intended as a first step to stimulate debate and discussion on persistence. It was recognized that there are a number of other issues that, due to time constraints, were not specifically included. These included the use and applicability of models (e.g., structure biodegradability relationships [SBRs] and quantitative structure biodegradability relationships [QSBRs]), which have been developed to predict biodegradation and abiotic processes. Although abiotic proceses (e.g., hydrolysis, oxidation, and photolysis) are recognized as key in assessing and determining persistence of many classes of chemicals in the environment, these were not specifically discussed as the workshop focused on biodegradation assessments. The workshop was by invitation and well attended from both a geographic and organizational perspective with 36 representatives from European regulatory bodies (AT, BE, CH, DE, DK, FI, FR, IT, LT, NL, NO, SE, UK), 17 from various industry sectors, 16 from academia (CH, DE, DK, ES, FR, NL, UK), and six attendees from consultancies and/or contract research organizations (CROs).

In the introductory session, it was highlighted that the industrial interest in product biodegradation assessments extends beyond simply regulatory compliance required to support both global and regional registration of products. Biodegradation data are used in applications other than REACH, for example, in schemes such as Environmentally Considerate Lubricants, EU Ecolabel criteria, and offshore applications. Credible and reliable data are essential to enable comparisons to be made between products and to develop “greener chemistry,” fulfilling customer and social demands.

Academics were keen to better understand the critical issues and relevant avenues of research to pursue, and how new research and scientific understanding could be utilized to improve product assessment, meet societal expectations, and improve environmental assessments. Finally, the regulatory community has the difficult task to meet societal expectations to demonstrate chemical safety while having to comply with rules and procedures before any amendments or significant changes could be made to existing regulations.

## WORKSHOP STRUCTURE

An overview of workshop sessions and technical presentations is provided in Table 1 with further details, including copies of the presentations, available on the Cefic LRI website (Cefic, 2019). After introductory talks to enable more focused discussions, the workshop was divided into three principal sessions on (1) the role of microbial communities in degradation testing (including adaptation, variability, growth, and cometabolism); (2) the impact of environmental factors on bioavailability and degradation; and, finally, (3) the interpretation of the OECD simulation test results and identified inherent challenges with these tests. Each of the focused sessions started with a series of presentations, followed by a question and answer (Q&A) session. The session chairs subsequently summarized the presentations and Q&A session with the objective of distilling key messages and proposals on how these could be either incorporated into and/or used to improve guidance.

**TABLE 1:** Overview of workshop sessions and technical presentations

Presentation title	Project details	Key presenter
Introductory session		
Persistence/biodegradation assessment from a regulatory point of view	Regulatory perspective	Vincent Bonnomet, European Chemicals Agency (ECHA)
Session 1:		
(1a) Role of microbial community in degradation testing		
The effect of including environmentally relevant microbial diversity in biodegradation screening tests for persistence assessment	Cefic LRI ECO 11	Russell Davenport, Newcastle University
Application of chemostat systems to include adaptation of microbial communities in persistency testing	Cefic LRI ECO 29	John Parsons, University of Amsterdam
(1b) Assessment of UVCBs		
Investigating mixture and concentration effects on biodegradation kinetics	DTU/Concawe	Rikke Hammershøj, Technical University of Denmark (DTU)
Session 2: Impact of environmental factors and bioavailability on degradation		
Identifying strategies that will provide greater confidence in estimating the degradation rates of organic chemicals in water, soil, and sediment	Cefic LRI ECO 31	Philipp Dalkmann, Bayer AG
Environmental risk assessment of poorly soluble substances: Improved tools for assessing biodegradation, (de)sorption, and modeling	Cefic LRI ECO 32	Fabio Polesel, DTU
Session 3: Interpretation of the OECD simulation test results and identified challenges		
Identifying limitations of the OECD water–sediment test and developing suitable alternatives to assess persistence	Cefic LRI ECO 18	Kathrin Fenner, Eawag
Limitations of OECD 307 and OECD 309 and recommendations for enhancements	Fraunhofer/Concawe	Dieter Hennecke, Fraunhofer IME
Biodegradation kinetics of hydrocarbons at low concentrations—Covering several orders of magnitude in hydrophobicity and volatility	DTU/Concawe	Heidi Birch, Technical University of Denmark (DTU)

*Note*: An initial presentation set out the workshop background and objectives. Each of the three sessions was followed by a brief presentation summarizing key messages and then an interactive Q&A discussion. Further details of the workshop agenda and presentations can be found on the Cefic LRI website (Cefic, 2019).

Abbreviations: Concawe, Conservation of Clean Air and Water in Europe; LRI, Long‐Range Research Initiative; OECD, Organisation for Economic Co‐operation and Development; UVCBs, substances of unknown or variable composition, complex reaction products, or biological materials.

In addition to the platform presentations, there was a poster session to broaden the participation and provide an opportunity for others to present new approaches and research. This session was interactive with delegates reviewing the posters on their potential relevance for persistence and environmental risk assessment. The posters can also be found on the Cefic LRI website (Cefic, 2019).

## INTRODUCTION SESSION

The first presentation provided background and set the objectives of the workshop, which were to improve communication and interactions between industry, academia, and the regulators regarding the current status of persistence assessments and recent scientific developments in the field. In terms of disseminating new findings, the intent was not just to provide updates on science but to consider how this new knowledge can

- be translated into sound advice and/or rules that are generally applicable to chemicals;
- lead to a better definition of the importance of new findings and/or insights and their influence on current persistence assessments;
- determine persistence criteria that represent sound science;
- clarify how findings interlink with other areas and/or activities such as research on bioavailability, formation of nonextractable residues (NERs), and so forth;
- be communicated in a transparent and credible way and easy‐to‐understand processes, enabling this to enhance guidance.

Next, an overview of the EU REACH regulation data requirements was presented, and a summary of the persistence data required is provided in Table 2. This presentation included some update on acceptability of modification in the enhanced ready biodegradability tests (RBTs), temperature specification for simulation tests, and NERs. As such, this presentation spanned areas of interest for all of the sessions and is consequently taken into account in the wrap‐ups for each session.

**TABLE 2:** Standard information requirements for (bio)degradation under current EU REACH regulations

Tonnage band (tons/year/registrant)	Required test data	Main tests used and comments
1–10	Ready biodegradability	OECD 301 series, OECD 306, and OECD 310
10–100	Ready biodegradability	As above
	Further information if CSA indicates the need, for example, substance screens a potential persistent, bioaccumulative, and toxic and/or vPvB	Methods depend on the need
100–1000	Ready biodegradability	As above
	Hydrolysis	For example, OECD TG 111: Hydrolysis as a function of pH
	Simulation of biodegradation in water^a^	OECD 309
	Simulation of biodegradation in sediment^b^	OECD 308
	Simulation of biodegradation in soil^c^	OECD 307
	Identification of degradation products^d^	
Over 1000	Ready biodegradability	As above
	Hydrolysis	As above
	Simulation of biodegradation in water^a^	As above
	Simulation of biodegradation in sediment^b^	As above
	Simulation of biodegradation in soil^c^	As above
	Identification of degradation products^d^	As above
	Further testing shall be proposed if chemical safety assessment indicates a need for additional data on the degradation of the substance	

Abbreviation: CSA, Chemical Safety Assessment; EU REACH, European Union Registration, Evaluation, Authorisation and Restriction of Chemicals; OECD, Organisation for Economic Co‐operation and Development; vPvB, very Persistent and very Bioaccumulative.

^a^ Not needed if the substance is highly insoluble in water and/or is readily biodegradable.

^b^ Not needed if the substance is readily biodegradable and/or direct and indirect exposure of sediment is unlikely.

^c^ Not needed if the substance is readily biodegradable and/or direct and indirect exposure of soil is unlikely.

^d^ Not needed if the substance has a low potential for bioaccumulation (for instance, a log*K*_ow_ < 3) and/or a low potential to cross biological membranes, and/or direct and indirect exposure of the aquatic compartment is unlikely.

The presentation clarified current ECHA guidance for RBTs, in which inocula derived from activated sludge or sewage effluent are recommended and modifications for improving the bioavailability of poorly water‐soluble substances (e.g., the use of silica gel or oil matrices, emulsifiers, or solvents) are permitted. However, increasing biomass concentration and diversity and low‐level preadaptation to the test item and the addition of cosubstrate(s) are not permitted.

Regarding inherent biodegradation tests, such as the Zahn–Wellens test (OECD 302B) and MITI II test (OECD 302C), these are not a standard information requirement under REACH; however, there is some guidance on how the data can be used. One fundamental point is that although pre‐exposure of the inoculum (preadaptation) is allowed in the test guidelines, this is not allowed for the P/vP assessment.

The reference temperature for the PBT and/or vPvB assessment and risk assessment has been set to 12 °C (285 K) for new studies. Furthermore, for any half‐lives that have been derived from simulation tests conducted at different temperatures, these should be extrapolated to 12 °C.

A summary of proposed revisions of the guidance on NERs, which was based on recommendations made by Kästner et al. (2018), was also provided. Ultimately, the intent is to differentiate total NER into different types according to their potential for remobilization, which requires using chemicals labeled with radioactive (for instance, ^14^C) or stable (for instance, with ^13^C and ^15^N) isotopes. A schematic of the proposed guidance is provided in the ECHA presentation and poster on the Cefic website (Cefic, 2019).

## SESSION 1

### Role of microbial community in degradation testing

The key message from this session was that standard biodegradation screening tests, often the first step in persistence assessments, are notoriously variable and, due to limitations in test designs and substance properties, are often considered to be unsuitable for assessing the biodegradability of many substances. These tests are designed to be stringent and have known limitations, which is why these form only one step of the ECHA integrated assessment and testing strategy (ITS) for persistence assessment (ECHA, 2017).

The known limitations were discussed in the first presentation in this session, and these relate mainly to inocula, variability, and reliability. It has been established that standard inocula preparations significantly reduce detectable diversity (*p* < 0.01) and select for nonpredominant taxa (Goodhead et al., 2013). As summarized by Kowalczyk et al. (2015), the tests use widely different inocula from environmental compartments with different microbial densities and diversities. These factors can partly explain the high coefficients of variation (typically 30%–54%) and high chance (20%–80%) of false negatives (i.e., when substances that are demonstrated and/or known to be readily biodegradable fail) seen in these tests (ECETOC, 2007; Kowalczyk et al., 2015).

Assessment of impacts of inocula in terms of both source and concentration of microbes undertaken in the Cefic LRI ECO 12 project, as reported by Martin et al. (2017), was extended to investigate potential improvements for other biodegradation screening tests, notably the OECD 306 test for biodegradability in seawater. The proposed improvements were based upon recommendations from a multistakeholder workshop (ECETOC, 2017; Ott et al., 2019) and assessed in an international ring test involving 13 laboratories. The main changes were that the proposed improved method incorporated increased bacterial cell numbers and ran beyond 60 days. The methods were considered to better represent the microbial diversity inherent in the sampled environments and, based on the biodegradation behavior of five reference chemicals, the new method is considered to be a more reliable and less variable method for assessing marine biodegradability (Ott et al., 2020).

In the next presentation, the significance of taking adaptation into account in persistence assessment was discussed. Consideration of adaptation in persistence assessments is viewed by many to be essential, and this concept has been around for some time. For example, Thouand et al. (1996) demonstrated that allowing adaptation can improve persistence assessments. More recently, adaptation of microbial communities present in ecosystems upon exposure of substance supporting growth has been described comprehensively (Poursat et al., 2019, 2020). This phenomenon is an important process involved in the biodegradation of naturally occurring chemicals and should somehow be taken into account in the assessment process.

In the workshop presentation, the case of *l*‐glutamate‐*N*,*N*‐diacetate (L‐GLDA), a phosphate replacement in automatic dishwashing detergents, was discussed. It was noted that the study by Itrich et al. (2015) systematically documented field adaptation of this new consumer product chemical across a large geographic region and confirmed the ability of laboratory simulation studies to predict field adaptation.

The Cefic LRI ECO 29 project presentation described how chemostat systems were employed to assess the effect of including adaptation of microbial communities in persistence testing. The influences of inocula source and pre‐exposure on the results of OECD 310 tests were demonstrated. Inocula source did have an impact on the results and pre‐exposure, for some chemicals (e.g., metformin and its metabolite guanylurea), was shown to significantly improve degradation and appeared to “smooth out” some of the variability in degradation rates observed for different inocula sources. The key conclusions were as follows: (1) At least for some chemicals, the biodegradation capacity of microbial communities increases due to adaptation to pollutants during long‐term exposure, resulting in faster biodegradation of otherwise persistent chemicals; (2) adaptation of microbial communities can be achieved under defined and relevant conditions in chemostat systems, although loss of competent microorganism may eventually take place; and (3) taking adaptation into account in testing protocols could result in a more realistic and reproducible assessment of biodegradability and persistence. However, this raised fundamental questions regarding the implementation of preadaptation in practical protocols for regulatory testing and how these data could be used in regulatory persistence assessment.

### Assessment of UVCBs

A new approach for assessing complex substances (UVCBs), notably petroleum products, was highlighted in the third presentation of the session. Further details of this research have been published by Hammershøj et al. (2019). Information requirements may be mitigated by using the hydrocarbon block (HCB) method as a framework to perform the environmental assessment. The HCB method resolves complex petroleum substances into pseudocomponents (“blocks”) that are defined by carbon number and hydrocarbon class (e.g., paraffins). However, fundamental questions regarding how representative constituents are for each block and how properties such as persistence vary within each block are currently left unanswered. Therefore, a project was initiated by Concawe to assess effects on biodegradation kinetics when (1) increasing test concentration of individual hydrocarbons and (2) undertaking biodegradation studies of single hydrocarbons in isolation and as mixtures of up to 16 hydrocarbon components.

The method utilized a new partitioning‐based platform for biodegradation testing where passive dosing was used to prepare the aqueous stock solution. The key findings from this research were that substance concentration affected biodegradation kinetics more than the number of mixture constituents being assessed. Consequently, simultaneous testing of multiple chemicals at low concentrations seems viable and can accelerate the generation of biodegradation kinetic data. Furthermore, these data are considered to be more environmentally relevant as compared with data from tests conducted with single chemicals at much higher concentrations.

### Summary of Session 1

An overview of the salient points from the presentations made in Session 1 has been summarized in Table 3. In the Session 1 summary and Q&A session, the following points and opinions were raised:

- One view was that growth‐linked degradation (i.e., degradation of chemicals used as sources of carbon, leading to increased populations of degrading microorganisms and in general to mineralization of the chemicals) has several advantages over cometabolic degradation. As such, greater emphasis should be placed on tests (experiments) detecting growth‐linked biodegradation in comparison to those also determining biodegradation through cometabolic transformation in persistence assessments.
- Environmental half‐lives of substances supporting growth do change constantly because the number of competent organisms (catalysts) varies with the availability of the substance. It was therefore suggested that rather than focus on assessing half‐lives, substances should be assigned to categories or bins.
- Robustness and applicability of tests (OECD 301 series and OECD 310) detecting growth‐linked biodegradation should be increased in a tiered assessment approach by allowing longer test periods, improved (e.g., more concentrated, diverse) inocula, and adaptation (pre‐exposure).
- Specific analysis is a useful tool for single components to clarify transformation to metabolites versus mineralization, as shown in the Cefic LRI ECO 29 project (Poursat et al., 2020). Furthermore, the Concawe biodegradation kinetics studies (Hammershøj et al., 2019) show that specific analysis in batch experiments is a useful tool to assess the nonpersistence of multiconstituents. Yet, it needs to be kept in mind that these provide information on the “rate” and occurrence of initial biotransformation, but not of mineralization per se.
- Environmentally relevant testing should be considered, that is, testing of constituents at low concentrations and in mixtures.
- Which research findings presented have potential to improve the current P assessment paradigm, and can priorities be identified?
- Further work is needed to implement the improvements, that is, can some practical and regulatory acceptable ways forward be identified?

**TABLE 3:** Session 1—Key points from session on role of microbial community (adaptation, variability, growth, and cometabolism) and assessing UVCBs

Need for more robust screening (enhanced) biodegradability tests	
Suggestion	Initial feedback
Use widely different fresh water inocula, more concentrated seawater inocula	Not widely accepted by regulators
	Need to improve and/or revise guidance? OECD 306 could have used enhanced inocula concentrations if reliable methods were available when original guidance was written^a^
Include adaptation as tool to improve screening test	Not widely accepted by regulators (e.g., not accepted under EU REACH), but need for discussion as adaptation can be a key removal mechanism
	Length and methods of exposure need to be environmentally relevant
	Assess influence on pre‐exposed inocula on simulation (e.g., OECD 309) test results
Use of specific analyses at low concentrations in an OECD 309 test setup	Required to assess UVCBs and multiconstituent substances
	The way forward may be to use in combination with ready biodegradation tests (RBTs) or enhanced test (mineralization)

Abbreviations: EU REACH, European Union Registration, Evaluation, Authorisation and Restriction of Chemicals; OECD, Organisation for Economic Co‐operation and Development; UVCB, substances of unknown or variable composition, complex reaction products, or biological materials.

^a^ As the workshop reference in March 2020 OSPAR has accepted the recommendations made by Ott et al. (2020) regarding biomass concentration for chemical persistence assessment B. Rowles, Cefas, personal communication.

It should be noted that, due to time limitations, the opinions expressed and latter questions could not be discussed or agreed on in detail. As such, they were simply identified as points warranting further post‐workshop discussion.

## SESSION 2: IMPACT OF ENVIRONMENTAL FACTORS AND BIOAVAILABILITY ON DEGRADATION

An overview of the Cefic LRI ECO 31 project was first presented. This project had two primary objectives: (1) To review state of the science on chemical degradation and persistence assessment and (2) to provide an evidence‐based evaluation of the key factors that drive chemical degradation rates. The investigation found that the main factors that drive atrazine degradation in laboratory studies are the same factors that drive atrazine degradation in the field, which were the atrazine application history and the soil texture. In addition, for other plant protection products with sufficient data available for the type of multivariable analysis used, again, chemical application history and also biomass concentrations were important factors. These case studies confirmed the utility of multivariable workflows for identifying key factors driving degradation half‐lives. However, it is recognized that when applied to the smaller datasets available for some other pesticides (and for other chemicals in general), this may lead to less reliable results. Furthermore, there may be other environmental factors that are important and responsible for variable degradation that cannot be considered because they have not been recorded in the respective degradation studies. Further work in this area is considered to be valuable, as the results of such metadata analyses can be used to inform the future evolution of OECD and similar guidelines to control for and record the most important environmental factors that contribute to the magnitude (and hence variability) of degradation rates. It is recognized that these guidelines may need to be malleable, as the role of each factor may depend also on intrinsic chemical properties.

The next presentation, based on the findings of the Cefic LRI ECO 32 project, gave an overview of the challenges of persistence assessment for poorly soluble substances (i.e., expected water solubility <<1 mg/L, log*K*_ow_ > 5.5). Low solubility raises challenges in the conduct and interpretation of OECD simulation tests (307–309). The same is also true when simulation tests are used to assess volatile substances. In the testing and analysis, problems are encountered due to their hydrophobic nature and strong sorption to surfaces, and establishing constant and measurable exposure concentrations is challenging. For these types of poorly soluble substances, it is also important to understand how bioavailability and intrinsic biodegradation potential act together to produce an observed persistence outcome.

The Cefic LRI ECO 32 project specifically investigated whether the aqueous biodegradation of poorly soluble substances can be reliably assessed, taking into account the influence of (de)sorption to and/or from solids (e.g., sediments) and, crucially, whether it is feasible to distinguish between bioavailability‐limited biodegradation (i.e., biodegradation where the rate is determined by quickly adsorbed and other inaccessible chemicals that become available for uptake by degrading microorganisms) and intrinsic biodegradation potential. The conclusion drawn from this research using radiolabeled dodecylbenzene (log*K*_ow_ = 8.65) and pyriproxyfen (log*K*_ow_ = 5.55) was that their biodegradation could be evaluated by combining novel testing methods and modeling work.

In terms of key messages regarding persistence assessment, it was concluded that the currently used persistence indicators (e.g., half‐lives), particularly in the case of poorly soluble substances, combine information on the intrinsic biodegradation potential of the substances with bioavailability limitations. Hence, simulation tests, such as the OECD 309 method, do not exclusively reflect the inherent biodegradation potential (i.e., recalcitrance of the molecule[s] and ability of bacteria to cleave molecular bonds).

### Summary of Session 2

An overview of the presentations regarding potential outcomes to improve persistence assessments and considerations for additional information and/or future research is provided in Table 4. These show that the approaches do have potential to resolve some of the issues currently encountered and, as discussed by workshop participants, some applications could be already used to improve risk assessments. Other points regarding the status and considerations for current OECD tests distilled from the Q&A session are as follows:

- The OECD tests do not adequately cover the range and diversity of environmental factors that are important for assessing degradation—How can this be resolved?
- More research and guidance are needed on the effects of exposure history, microbial community biomass and/or composition, soil properties, and so forth, on test outcomes.
- As it is unlikely that all variability of testing outcomes can be removed, how can the issue of inherent variability be redressed?
- Biodegradability indicators (e.g., half‐lives) derived from current simulation tests may not adequately describe the inherent biodegradation potential of poorly soluble substances due to influence of bioavailability limitations.
- Could the above point be mitigated by considering a risk‐based approach, that is, by explicitly accounting for bioavailability and/or activity?
- A potential improvement is to combine testing methods with inverse modeling (i.e., developing models for key chemical and test system properties to assess how these affect actual vs. anticipated biodegradation) to disentangle biodegradation and partitioning to yield compartment‐specific and bioavailability‐normalized indicators of biodegradability.

**TABLE 4:** Session 2—Key points from session on impact of environmental factors and bioavailability on degradation

Project	Potential to improve current P assessment	Further requirements and research needs
General considerations		
Cefic LRI ECO 31	Could such statistical models be used to predict, scale, or weight half‐lives?	Application of the workflow to other environmental compartments
Identifying strategies that will provide greater confidence in estimating the degradation rates of organic chemicals in water, soil, and sediment	Guidance on minimum environmental reporting is required for all simulation tests and collated into a publicly available database	Is a better process and/or mechanistic understanding of these statistical associations warranted?
		What other environmental parameters need to be included?
		How could this information be used in a regulatory context?
Extension of chemical applicability domain to volatile and hydrophobic chemicals		
Cefic LRI ECO 32	Consider using passive dosing and/or sampling and modeling to delineate bioavailability limitation from true biodegradation half‐lives for poorly soluble chemicals	Verification of proposed method with further chemicals and at further laboratories
Environmental risk assessment of poorly soluble substances: Improved tools for assessing biodegradation, (de)sorption, and modeling	Use of cell quantification	Need to define cell quantification methods and ensure these are amenable to other laboratories

Abbreviation: LRI, Long‐Range Research Initiative; P, persistence.

As with the previous session, the question of how to move forward and maximize the value of the recent research hinges on the acceptance of the new approaches. There is clearly a role in persistence assessment for some of the proposed strategies, but they are new, some require more data than currently available for the majority of chemicals (may be applicable for plant protection products), and there would naturally be concerns regarding the need to verify and obtain regulatory approval and/or endorsement of the proposed improvements.

## SESSION 3: INTERPRETATION OF SIMULATION TESTS

Simulation tests (e.g., OECD 307, 308, 309, and 314) are higher tier tests required if a substance cannot be classified as readily biodegradable based on RBT (which also includes approved enhanced RBTs) tests. Simulation tests are meant to provide information for regulatory risk assessment on the transformation or mineralization half‐lives in one or several environmental compartments for persistence assessment and exposure modeling, and to assess the identity and quantity of transformation products.

It was noted from the introductory sessions (and Table 2) that in its current guidance, ECHA does not use the OECD 314 series of tests to derive environmental half‐lives for PBT assessment. However, such tests provide valuable information for informing the persistence assessment of down‐the‐drain chemicals, the vast majority of which should be treated in wastewater treatment plants before entering the environment. Agreement is required on how such data can be used to assess environmental persistence; however, one option would be as part of weight of evidence arguments, as these are OECD‐approved tests and, therefore, should be considered to be credible.

An overview of the simulation tests and issues associated with them identified in the Cefic LRI ECO 18 project were presented. Results of this project are reported in Honti and Fenner (2015), Honti et al. (2016), and Shrestha et al. (2016). One of the key experimental issues with the OECD 308 test is that it requires a significant amount of experimental effort (employs ≥ 60 vessels; requires labeled compounds) and is expensive. It also uses a high sediment:water ratio, thereby shifting mass distribution excessively toward sediment. As a consequence, excessive sorption may lead to reduced bioavailability, and hence effectively “mask” degradation; redox gradients may occur within the sediment layer; and, in many cases, extensive NER formation is observed. As such, because high sediment:water ratios are not typical of most surface water bodies with relevant chemical input, it has been questioned whether the results of OECD 308 studies can be considered environmentally representative.

In terms of interpreting results from OECD 308 studies, there is dynamic partitioning of the chemicals between solid aerobic and/or anaerobic phase and water during incubation. Consequently, DT_50_ (the time within which the concentration of the test substance is reduced by 50%) water and DT_50_ sediment confound degradation and phase transfer, making those endpoints unsuitable for comparison to P cut‐off values or for exposure modeling. The total system DT_50_ is a better measure of degradation for sure, but it depends to some extent on the sediment:water ratio, as explained above, and hence is not fully test system‐independent. A TOC‐ and bioavailability‐normalized rate constant k’_bio_, which can be obtained through inverse modeling of data from OECD 308 studies, has been suggested to alleviate the above‐mentioned problems and to be a potentially useful indicator of the inherent biotransformation potential of the chemical by the given environmental microbial community.

Regarding OEC 309, the current guidelines allow for a large degree of freedom in experimental design (e.g., amount of sediment, stirred and/or shaken, light and/or dark, sediment sampling), leading to high variability in test outcomes. Further standardization of the test method seems warranted and a modified OECD 309 (with increased sediment concentration) was recommended as a simple, representative system to test biotransformation at the water–sediment interface.

With respect to further research, the need to improve assessment of NER was noted (it should be noted that this is being developed under the Cefic LRI ECO 24 and ECO 25 projects) as well as the need to develop and validate improved methods to measure active biomass. Finally, there should be further validation of conceptual soundness and applicability of k’_bio_ values with additional datasets.

The next presentation, based on research undertaken by the Fraunhofer IME for both Concawe and Cefic, identified the limitations and potential enhancements of the OECD 307 and OECD 309 tests. Some of the issues and recommendations for improvement for these tests have subsequently been published (Shrestha et al., 2019, 2020). For example, both tests are considered to be poorly suited for the assessment of volatile substances. However, in OECD 307, the use of closed systems improves mass balances and enables the quantification of volatilized fractions, but care is required to avoid oxygen depletion. Furthermore, interpretation is complicated due to competition between sorption, degradation, and volatilization. Main conclusions from this research project were as follows:

- OECD guidelines are often applied outside their scope (i.e., applicability domain), particularly, regarding substance properties. In such cases, using the standard test setup may lead to false data and results.
- A specific test setup has been developed, which has been shown to enable a complete mass balance for the OECD 307 test.
- O_2_ monitoring in closed flask tests is essential. It raises a new challenge, but improved optical sensors and technology are available.
- For OECD 309, further research is needed. Current setup and existing OECD technical guidance are not satisfactory.
- ^14^C‐labeled test substance avoids wrong interpretation and enables pathways to be shown.
- For upcoming NER assessment, ^14^C label is necessary.
- The proposed extended model considers volatilization. It is simple and pragmatic for generating the degradation kinetics with a good fit.

The final presentation in this session, based on research undertaken by the Danish Technical University for Concawe, highlighted a new approach using modified tests to assess biodegradation kinetics of hydrocarbons. These tests were designed to assess persistence of hydrocarbons (whose hydrophobicity and volatility spanned several orders of magnitude) at low concentrations. The new experimental platform developed for biodegradation measurements had similarities to (but was not the same as) OECD 309 simulation tests. It used environmentally relevant concentrations (ng–µg/L), environmentally native microorganisms, and closed test systems applicable to (semi)volatile chemicals.

The new platform was intended to be applicable for assessing nonlabeled substances (monitoring primary biodegradation), multiconstituent mixtures, and hydrophobic and volatile chemicals. This research has also been summarized in recent publications (Birch, Andersen, et al., 2017; Birch, Hammershøj, et al., 2017; Birch et al., 2018). The advantage of this system was that it enabled testing multiconstituent mixtures and yielded large sets of well‐aligned data. The method is based on substrate depletion and, therefore, only provides data on primary degradation. The current design is limited to aqueous media and there are concerns that the sample volume of 13.5 ml could be insufficient at low degrader densities. As in previous research, one of the critical issues is the acceptability of such data in a regulatory context. Although existing tests have been shown to be unsuitable for these substances, there is a need to gain approval regarding both the suitability and reliability of data generated with the newly proposed method to fulfill existing regulatory criteria.

### Summary of Session 3

Some potential considerations from Session 3 have been summarized in Table 5. It is recognized that improving study reproducibility, reliability, and comparability will allow consistent comparison for benchmarking and interpretation of persistence data. There is an identified need for robust modified studies to support and help interpret current OECD tests, especially for multiconstituent and/or UVCB testing. This is because the OECD tests are not considered to be “fit for purpose” for many chemical classes. Furthermore, although there is still a regulatory focus on half‐lives, new measures such as the TOC‐ and bioavailability‐normalized k’_bio_ indicator could provide additional information on the inherent biodegradation potential of chemicals and be used directly in exposure modeling.

**TABLE 5:** Session 3—Key points from session on issues with the interpretation of OECD simulation test; suggestions, potential to improve persistence assessments, and future activities

Main suggestions	Potential to improve current P assessment	Further requirements and research needs
Reproducibility and/or comparability of test outcomes		
Need for further standardization (and reporting) of experimental options, particularly in OECD 309	Reduced variation in test outcomes for individual substances	More experience with different possible OECD 309 settings
Testing multiconstituent mixtures yields large sets of well‐aligned data for multiple substances	Consistency between test outcomes for multiple substances	Feedback on problems and/or learnings encountered in Concawe projects (e.g., closed setup required for volatiles led to very slow degradation, even of reference compound)
	Potential to assess relative biotransformation potential among substances	
	Solid basis for comparison against benchmark chemicals or test‐specific P criterion	
Extension of chemical applicability domain to volatile and hydrophobic chemicals		
DTU/Concawe: Passive dosing w/loaded silicone rods; closed tests; sampling with SPME	(No) need for ^14^C‐labeled substances to ensure mass balance^a^	Maintenance of oxic conditions in closed tests with soil, sediment, and sludge
Fraunhofer/Concawe: Closed tests with O_2_ monitoring and tenax as sink for volatile fraction	Enables testing of multiconstituent mixtures	Tenax potentially too strong sink in OECD 309? Alternate test setup needed for highly volatile chemicals
	Coverage of high *K*_ow_ and *K*_aw_ substances	
	Broader range of chemicals amenable to P assessment	Reduced community diversity in small test volumes?
Normalization of half‐lives (or rate constants) to obtain robust descriptors of biotransformation potential		
ECO 18 has shown that correction for biomass and bioavailable concentrations yields substance‐specific biotransformation measure that is valid across different OECD 308 and 309 setups	Assessment of biotransformation potential becomes less dependent on test system (factoring out different degrees of sorption)	Robust and reproducible method to measure total active biomass required
	Potential for reduced testing requirements	Importance of differences in inocula versus functional saturation in natural communities
DTU/Concawe research indicates that correction for effective aqueous concentrations reveals higher rate constants	Potential for development of improved predictive models (e.g., quantitative structure biodegradability relationships, read‐across)	

Abbreviations: Concawe, Conservation of Clean Air and Water in Europe; DTU, Technical University of Denmark; OECD, Organisation for Economic Co‐operation and Development); P, persistence; SPME, solid‐phase microextraction.

^a^ The need for a complete mass balance is removed when determining primary degradation because results are expressed not as disappearance of the absolute or total amount of test chemical, but as disappearance relative to an abiotic control. However, to measure more than primary degradation (e.g., mineralization, nonextractable residue formation, etc.), a mass balance using radiolabeled chemicals would be required.

## POSTERS

Many of the posters provided supplementary information to support the presentations. A summary of the titles and authors is provided in Table 6 and actual presentations are available on the Cefic LRI website (Cefic, 2019).

**TABLE 6:** Summary of poster presentations

Authors	Title	Short synthesis
Redman et al.	Application of GC × GC to characterize biodegradation of crude oil using the hydrocarbon block method	Advanced 2D gas chromatography can be applied for determining half‐lives of major aliphatic and aromatic chemical classes and carbon numbers in complex products. Blockwise half‐lives were similar to available half‐lives for representative single constituents 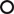
Schäffer et al.	Characterization of different NER types. NER and PBT assessment	Three NER types can be experimentally quantified: I sequestered (releasable), II covalent (hardly releasable), III biogenic. Type I NER is relevant for persistence assessment. Modeling (MTB) can be used to estimate the formation of biogenic NER 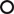 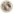
Ott et al.	Findings from an international ring test for an improved marine biodegradation screening test	Modification of OECD 306 (more bacterial cells and longer test duration) leads to more accurate and reliable persistence assessment by inclusion of extended lag phases and better representation of bacterial diversity in environmental matrices 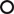
ECHA	Integrated testing strategy for persistency	The updated, revised Integrated Assessment and Testing Strategy (ITS) is necessary to conclude on the persistence/nonpersistence of substances, screening information → decision not P, not vP; for potential P/vP substances higher tier information is needed. The update also considers “difficult” substances (UVCB, impurities, additives, …) 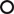
Hughes et al.	Persistence assessment of phenanthrene: A case study	In contrast to phenanthrene SVHC dossier (“is persistent”), presented OECD tests 301, 307, 308 indicate that phenathrene “is not persistent.” Bioavailability is of similar importance for biodegradability as experimental conditions (O_2_, inoculum, nutrients, …)
Bonnomet et al.	Steps needed for incorporating scientific developments into regulatory practice	Provides PBT/vPvB assessment guidance: Weight of evidence tools, difficult‐to‐test substances, use of QSAR, NER, interpretation of bioaccumulation data 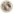
Nicholls et al.	Temperature and exposure history strongly influence GEO biodegradation in groundwater	Biodegradation of gasoline ether oxygenates (GEO) like MTBE depends on temperature (not Arrhenius‐like) due to T‐sensitivity of degraders and differs at uncontaminated and contaminated sites due to adaptation of degraders (gene copy numbers tested) 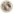
Sjøholm et al.	Temperature dependence of biodegradation kinetics in environmental surface waters and biodegradation testing	Temperature dependence (both of test conditions and original inoculum temp) of biodegradation kinetics for 30 chemicals and impact of test volume (# of degraders) can be tested by passive dosing 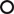
Mayer et al.	UVCB fate‐directed toxicity testing and risk assessment (UVCBFATETOX)–Cefic LRI ECO 42	Toxicity and bioaccumulation tests of persistent UVCB constituents can be determined by passive dosing at environmental relevant concentrations to develop an integrated risk assessment strategy of such complex products 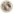
Lot et al.	Effluent biodegradability evaluation using whole effluent approach	Whole wastewater effluent approach: How to assess persistence (test only biodegradation potential or representative environmental conditions)? Which inoculum? Compare effluent toxicity before and after biodegradation rather than just testing biodegradation 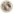

Abbreviations: ECHA, European Chemicals Agency; LRI, Long‐Range Research Initiative; MTB, Microbial Turnover to Biomass; MTBE, Methyl tert‐butyl ether; NER, nonextractable residue; OECD, Organisation for Economic Co‐operation and Development; PBT, persistent, bioaccumulative, and toxic; QSAR, Quantitative structure‐activity relationship; SVHC, substance of very high concern; UVCB, substance of unknown or variable composition, complex reaction products, or biological materials; vPvB, very Persistent and very Bioaccumulative.

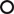
 Five or more participants felt ideas could support P assessments in the near term (i.e., methods, tools, and data are available).

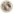
 Five or more participants felt ideas could support P assessments in the future (i.e., validation of approach/methods required).

## CONCLUSIONS

The workshop and parallel poster sessions presented recent research and suggested some practical approaches where these new ideas could be considered for incorporation into test guidelines and regulatory guidance. The current lack of consensus in some areas raises the potential for differing interpretation of available datasets with regard to persistence. These issues need to be openly discussed and where decisions are deemed to be scientifically questionable and a clear divergence of views is apparent, a mechanism needs to be established, so these can be appropriately resolved. This is of even greater significance if persistence is given more emphasis in chemicals assessment and decision‐making as proposed by some scientists (Cousins et al., 2019).

There are new challenges raised by different classes of substances, but it is possible to overcome these by modification of existing test methods. However, from a broader and specifically regulatory perspective it is important to raise awareness that the currently available OECD guidelines and methods might (in some cases will) not work for certain groups of chemicals without modifications. In that case, new scientific techniques and approaches must be considered to ensure persistence can be properly assessed.

There is a need for a robust chemical registration process in which all classes of chemicals can be assessed and if new assessment methods and/or approaches are proposed, they need to be able to withstand scrutiny. The challenge is to provide alternatives and improvements to the current testing paradigm, which can ultimately be accepted within the regulatory domain to increase confidence that the persistence properties of chemicals are correctly classified.

This workshop was seen as an important first step in identifying opportunities to try and improve understanding of factors affecting biodegradation assessments and better understanding of the persistence of chemicals (particularly UVCBs). A critical component is the interactions between scientists from different backgrounds to assess the current status of the science and gain consensus on how new knowledge can enhance regulatory systems. The emphasis of these engagements should be to develop consistency, identify and reach agreement on how new weight of evidence approaches for more challenging substances can be considered, and finally, identify any additional research required to enhance persistence assessments.

Subsequent to the workshop, there have been a number of key steps taken as part of this journey. In addition to the research being published, ECETOC has set up a multistakeholder persistence task force whose terms of reference align with those arising from the workshop (ECETOC, 2019). This task force has prepared a review on scientific concepts and methods to improve P assessments (R Davenport, Newcastle University, personal communication) and proposed a conceptual weight of evidence framework for P assessment (A Redman, ExxonMobil, personal communication). Furthermore, a new Cefic LRI research project ECO 52 was initiated with a call for proposals to expand the conceptual principles and applicability domain of persistence screening and prioritization frameworks, including single constituents, polymers, and UVCBs. The project team is currently working on three themes to deliver a step change in persistence assessments addressing bioavailability, complex substances, and overall persistence (Cefic, 2020). This ECO 52 project has a multistakeholder monitoring team to maximize input from experts in the regulatory, academic, and industrial communities. The final related activity is a planned second follow‐up Cefic LRI–Concawe–ECETOC joint workshop intended to be held in Helsinki in 2021.

In conclusion, the workshop was seen as the beginning of a long but important journey by acting as a catalyst to bring together expertise with the intention to enhance the scientific knowledge and guidelines to better understand the environmental persistence of chemicals.

## CONFLICT OF INTEREST

The authors declare that there are no conflicts of interest.

## Data Availability

This is a workshop summary and copies of presentations can be accessed via the Cefic LRI website (https://cefic‐lri.org/events/cefic‐lri‐concawe‐workshop‐on‐recent‐developments‐in‐science‐supportive‐to‐the‐persistencebiodegradation‐assessment/). Requests for updates are available from corresponding author Eleni Vaiopoulou, eleni.vaiopoulou@concawe.eu.
